# Supplementary material for: Effects of Acute 2,3,7,8-Tetrachlorodibenzo-p-Dioxin Exposure on the Circulating and Cecal Metabolome Profile
Source: Int J Mol Sci. 2021 Oct 30;22(21):11801. doi: 10.3390/ijms222111801 (PMC8583798; doi:10.3390/ijms222111801)
Supplement: Supplementary file 1 [file ijms-22-11801-s001.zip › ijms-1444410-supplementary.pdf]

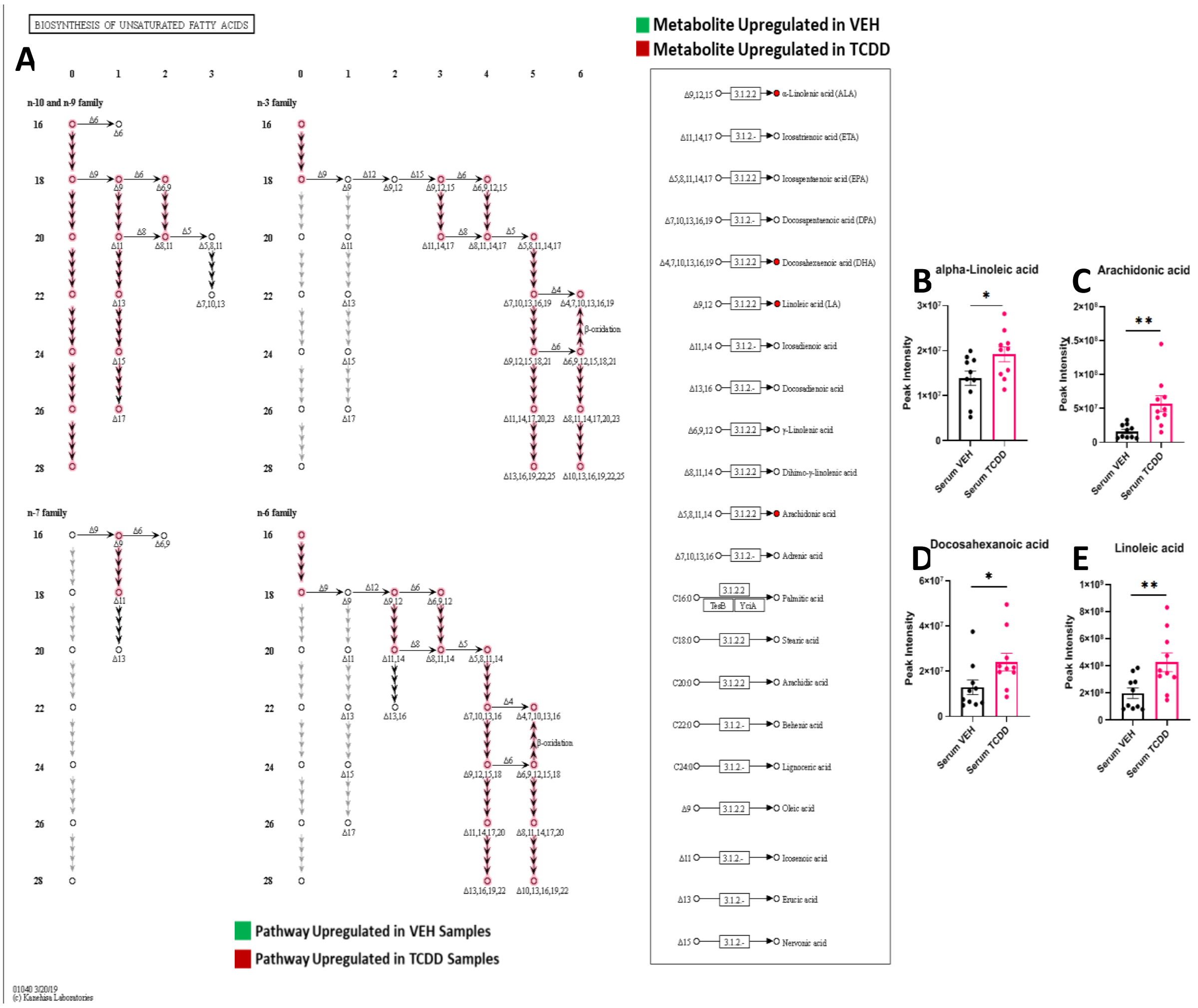

Figure S1. Biosynthesis of Unsaturated Fatty Acids in the Serum. (A) Biosynthesis of unsaturated fatty acid KEGG pathway illustrating metabolic networks including metabolites denoted by circles and associated enzymes denoted by squares. Significant metabolites are indicated by a solid node that correlates with upregulation in the TCDD (Red) or VEH (Green) exposed mice. Pathway sections and components highlighted by a red outline indicate likely enzymes and additional metabolites that may be involved in the difference of expression. (B-E) Bar charts demonstrating abundance of significantly modulated metabolites identified via the Biosynthesis of Unsaturated Fatty Acids KEGG pathway.

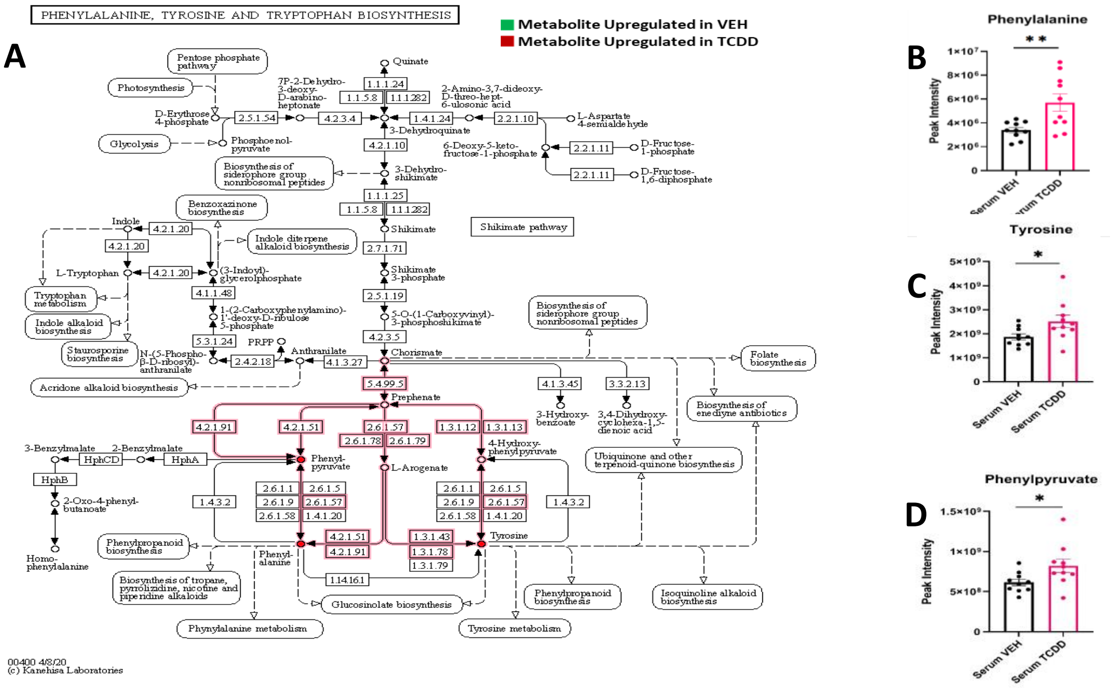

Figure S2. Phenylalanine, Tyrosine, and Tryptophan Biosynthesis in the Serum. (A) Phenylalanine, tyrosine, and tryptophan biosynthesis KEGG pathway illustrating metabolic networks including metabolites denoted by circles and associated enzymes denoted by squares. Significant metabolites are indicated by a solid node that correlates with upregulation in the TCDD (Red) or VEH (Green) exposed mice. Pathway sections and components highlighted by a red outline indicate likely enzymes and additional metabolites that may be involved in the difference of expression. (B-C) Bar charts demonstrating abundance of significantly modulated metabolites identified via the Phenylalanine, Tyrosine and Tryptophan Biosynthesis KEGG pathway.

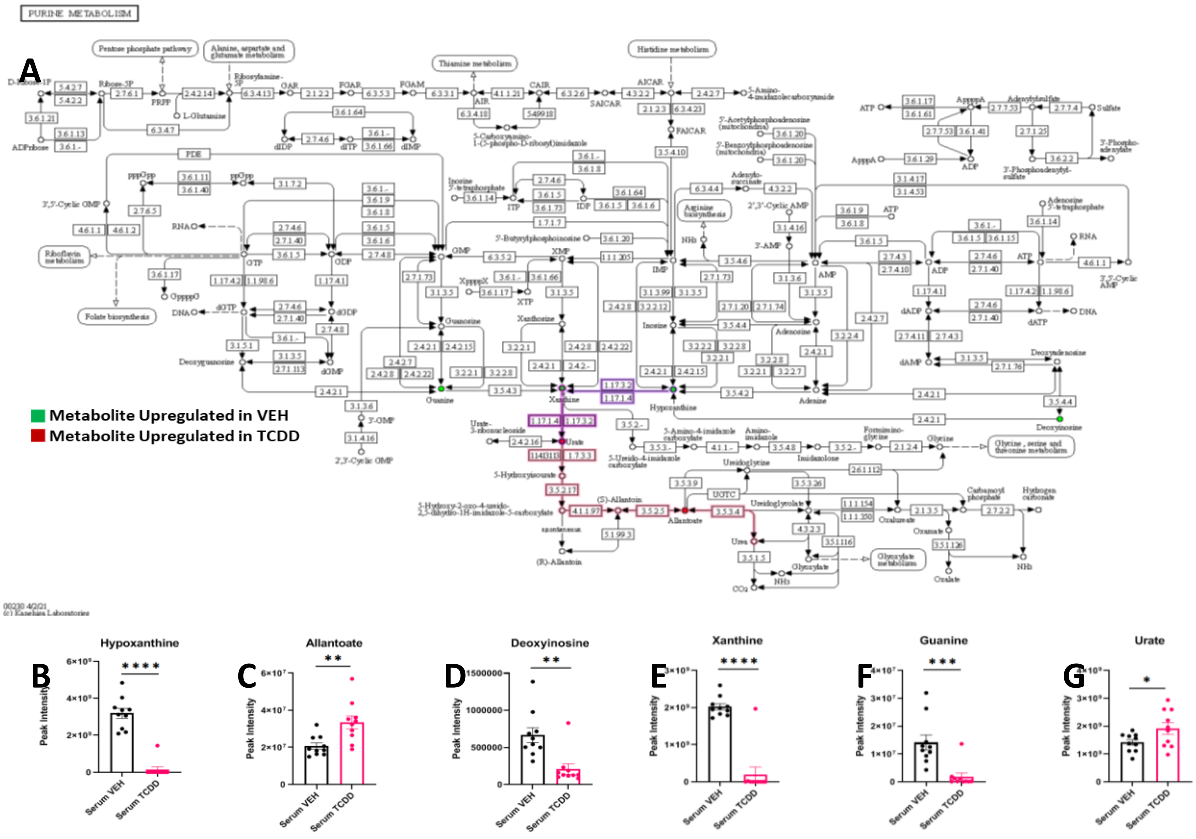

Figure S3. Purine Biosynthesis in the Serum. (A) Purine metabolism KEGG pathway illustrating metabolic networks including metabolites denoted by circles and associated enzymes denoted by squares. Significant metabolites are indicated by a solid node that correlates with upregulation in the TCDD (Red) or VEH (Green) exposed mice. Pathway sections and components highlighted by a red outline indicate likely enzymes and additional metabolites that may be involved in the difference of expression. (B-G) Bar charts demonstrating abundance of significantly modulated metabolites identified via the Purine Biosynthesis KEGG pathway.

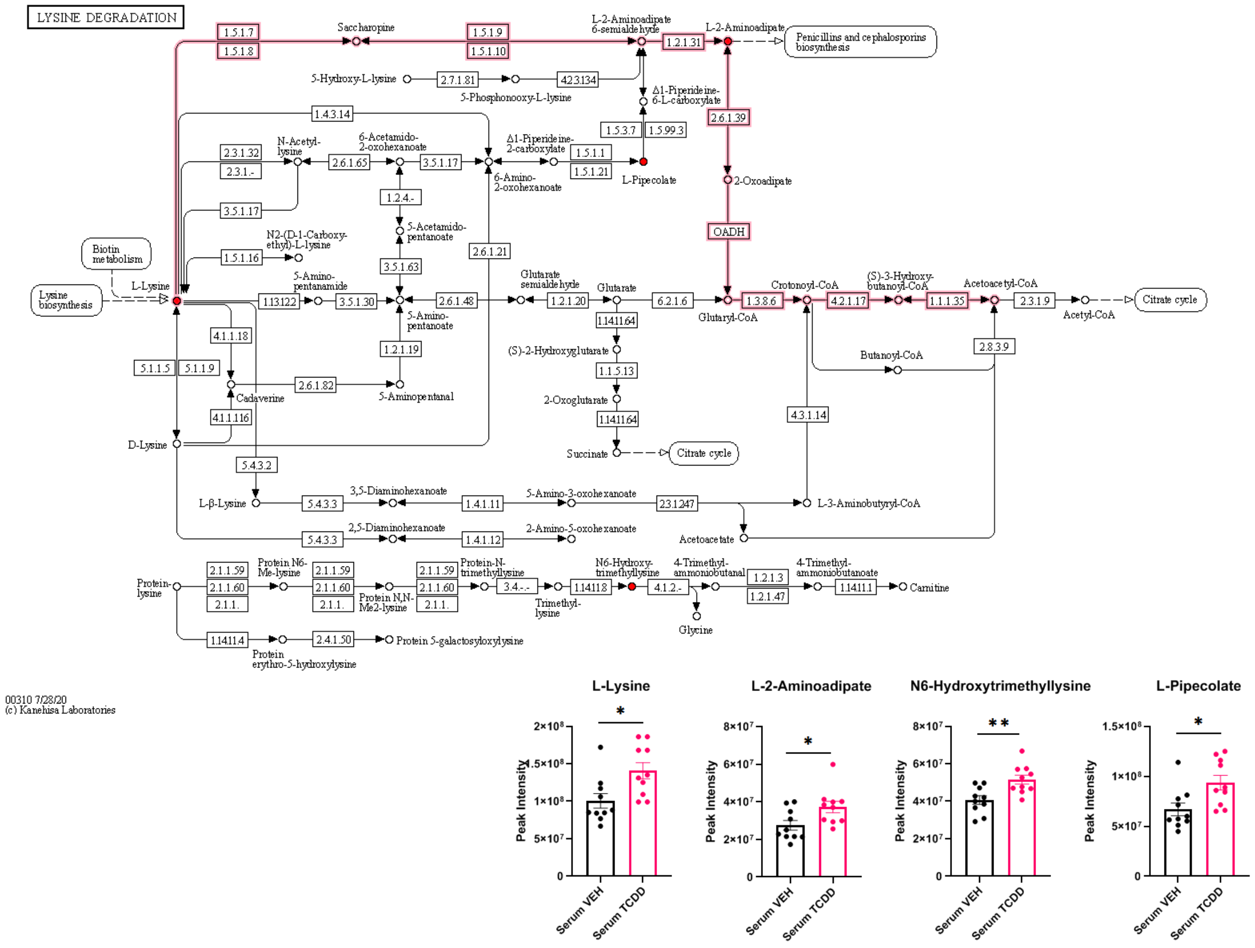

Figure S4. Lysine Degradation in the Serum. (A) Lysine degradation KEGG pathway illustrating metabolic networks including metabolites denoted by circles and associated enzymes denoted by squares. Significant metabolites are indicated by a solid node that correlates with upregulation in the TCDD (Red) or VEH (Green) exposed mice. Pathway sections and components highlighted by a red outline indicate likely enzymes and additional metabolites that may be involved in the difference of expression. (B-E) Bar charts demonstrating abundance of significantly modulated metabolites identified via the Lysine Degradation KEGG pathway.

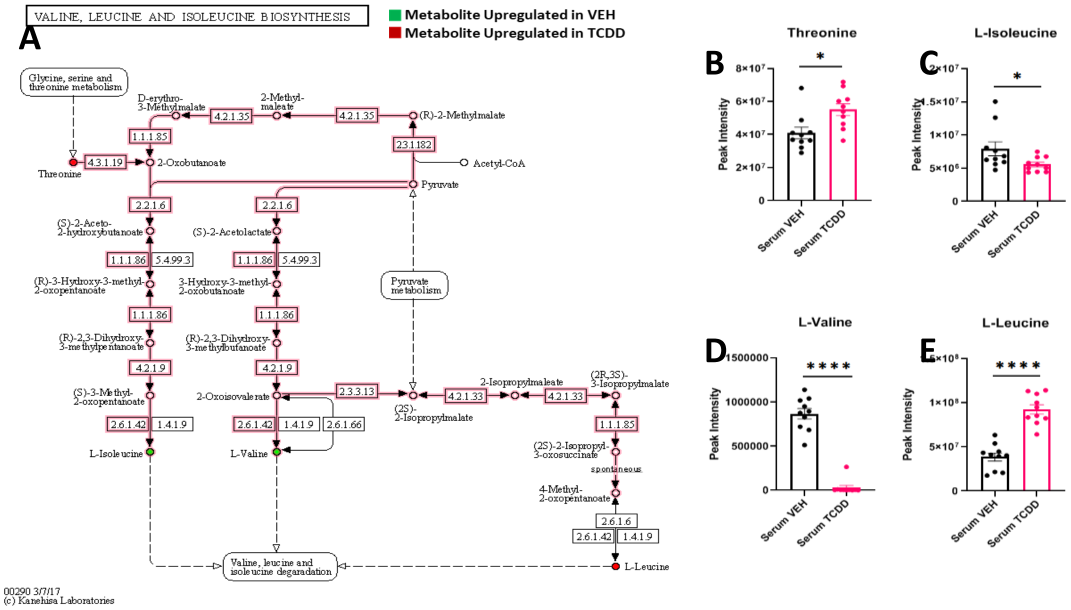

Figure S5. Valine, Leucine and Isoleucine Biosynthesis in the Serum. (A) Valine, leucine, and isoleucine biosynthesis KEGG pathway illustrating metabolic networks including metabolites denoted by circles and associated enzymes denoted by squares. Significant metabolites are indicated by a solid node that correlates with upregulation in the TCDD (Red) or VEH (Green) exposed mice. Pathway sections and components highlighted by a red outline indicate likely enzymes and additional metabolites that may be involved in the difference of expression. (B-E) Bar charts demonstrating abundance of significantly modulated metabolites identified via the Valine, Leucine and Isoleucine Biosynthesis KEGG pathway.

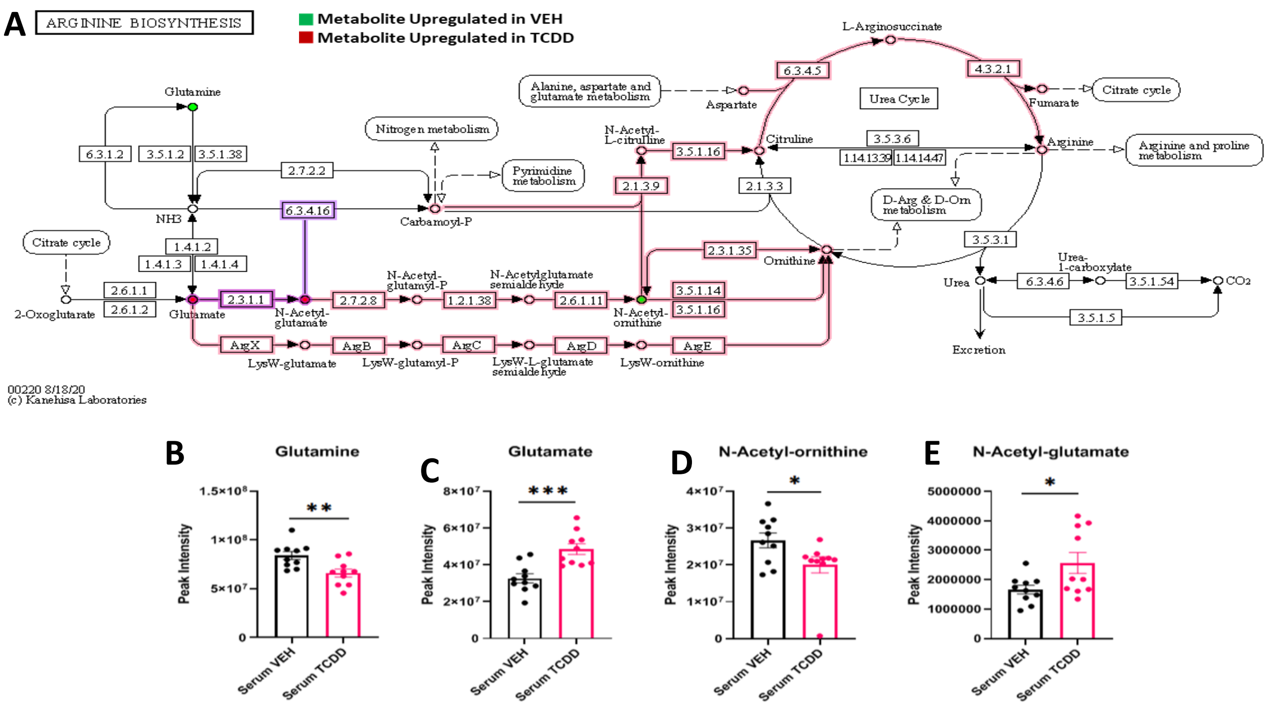

Figure S6. Arginine Biosynthesis in the Serum. (A) Arginine biosynthesis KEGG pathway illustrating metabolic networks including metabolites denoted by circles and associated enzymes denoted by squares. Significant metabolites are indicated by a solid node that correlates with upregulation in the TCDD (Red) or VEH (Green) exposed mice. Pathway sections and components highlighted by a red outline indicate likely enzymes and additional metabolites that may be involved in the difference of expression. (B-E) Bar charts demonstrating abundance of significantly modulated metabolites identified via the Arginine Biosynthesis KEGG pathway.

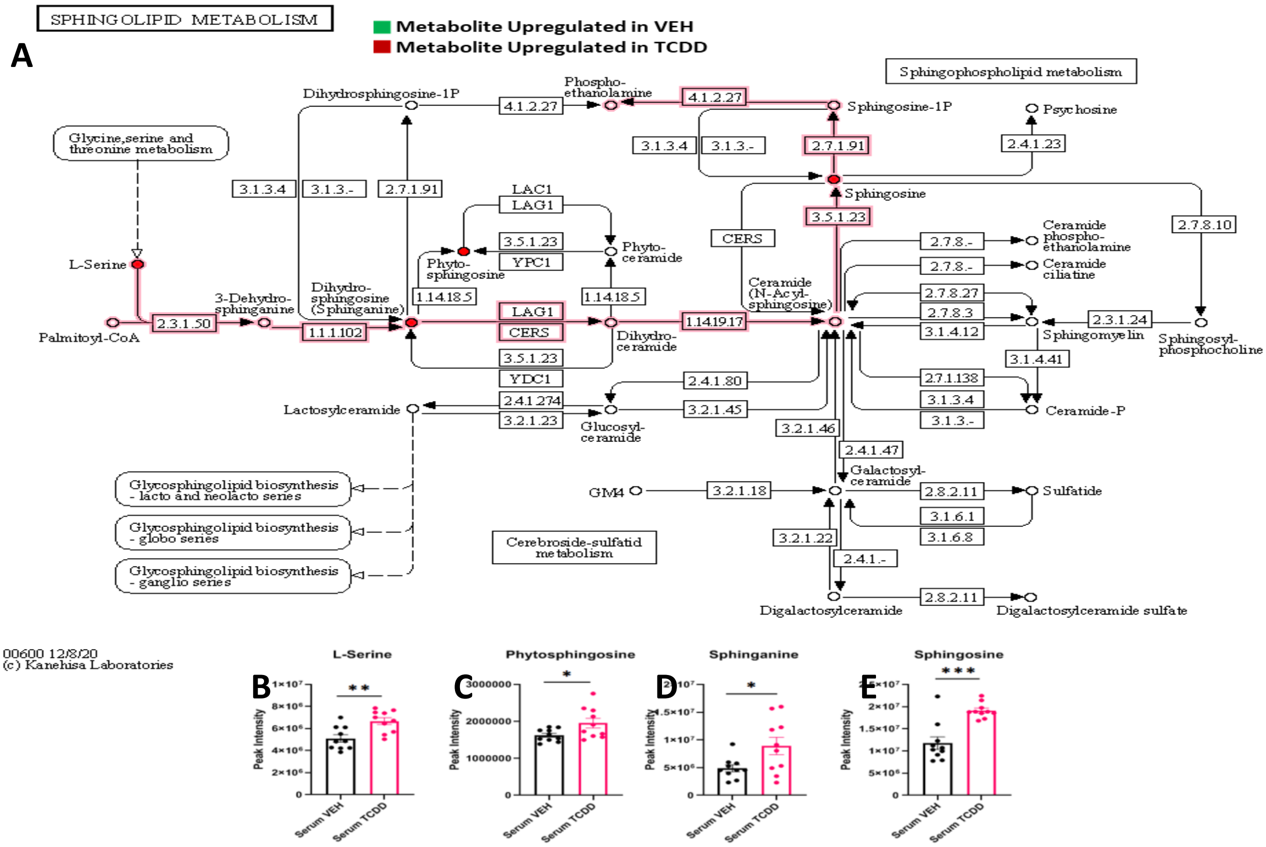

Figure S7. Sphingolipid Metabolism in the Serum. (A) Sphingolipid metabolism KEGG pathway illustrating metabolic networks including metabolites denoted by circles and associated enzymes denoted by squares. Significant metabolites are indicated by a solid node that correlates with upregulation in the TCDD (Red) or VEH (Green) exposed mice. Pathway sections and components highlighted by a red outline indicate likely enzymes and additional metabolites that may be involved in the difference of expression. (B-E) Bar charts demonstrating abundance of significantly modulated metabolites identified via the Sphingolipid Metabolism KEGG pathway.

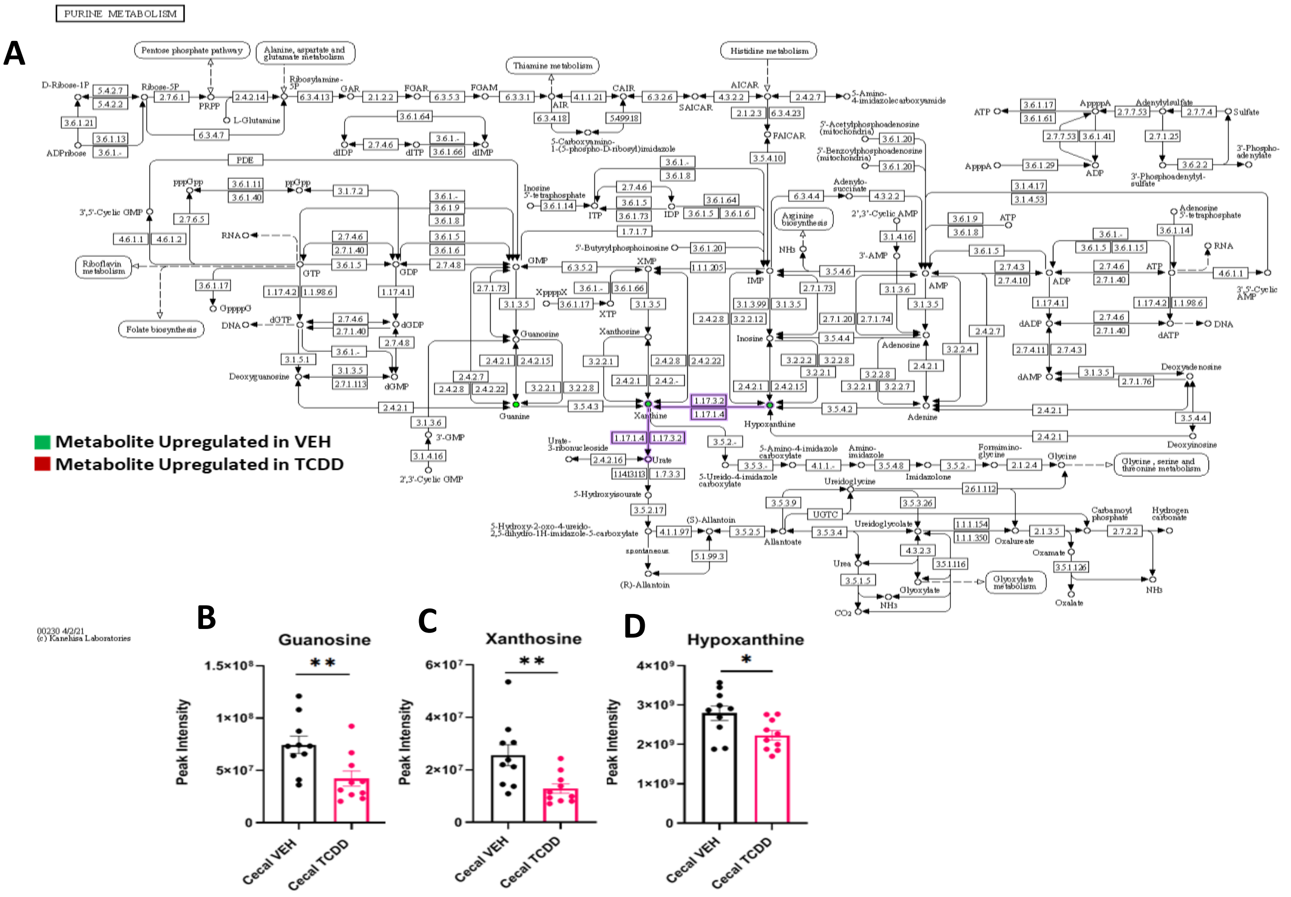

Figure S8. Purine Metabolism in the Cecal Contents. (A) Purine metabolism KEGG pathway illustrating metabolic networks including metabolites denoted by circles and associated enzymes denoted by squares. Significant metabolites are indicated by a solid node that correlates with upregulation in the TCDD (Red) or VEH (Green) exposed mice. Pathway sections and components highlighted by a red outline indicate likely enzymes and additional metabolites that may be involved in the difference of expression. (B-D) Bar charts demonstrating abundance of significantly modulated metabolites identified via the Purine Metabolism KEGG pathway.

A

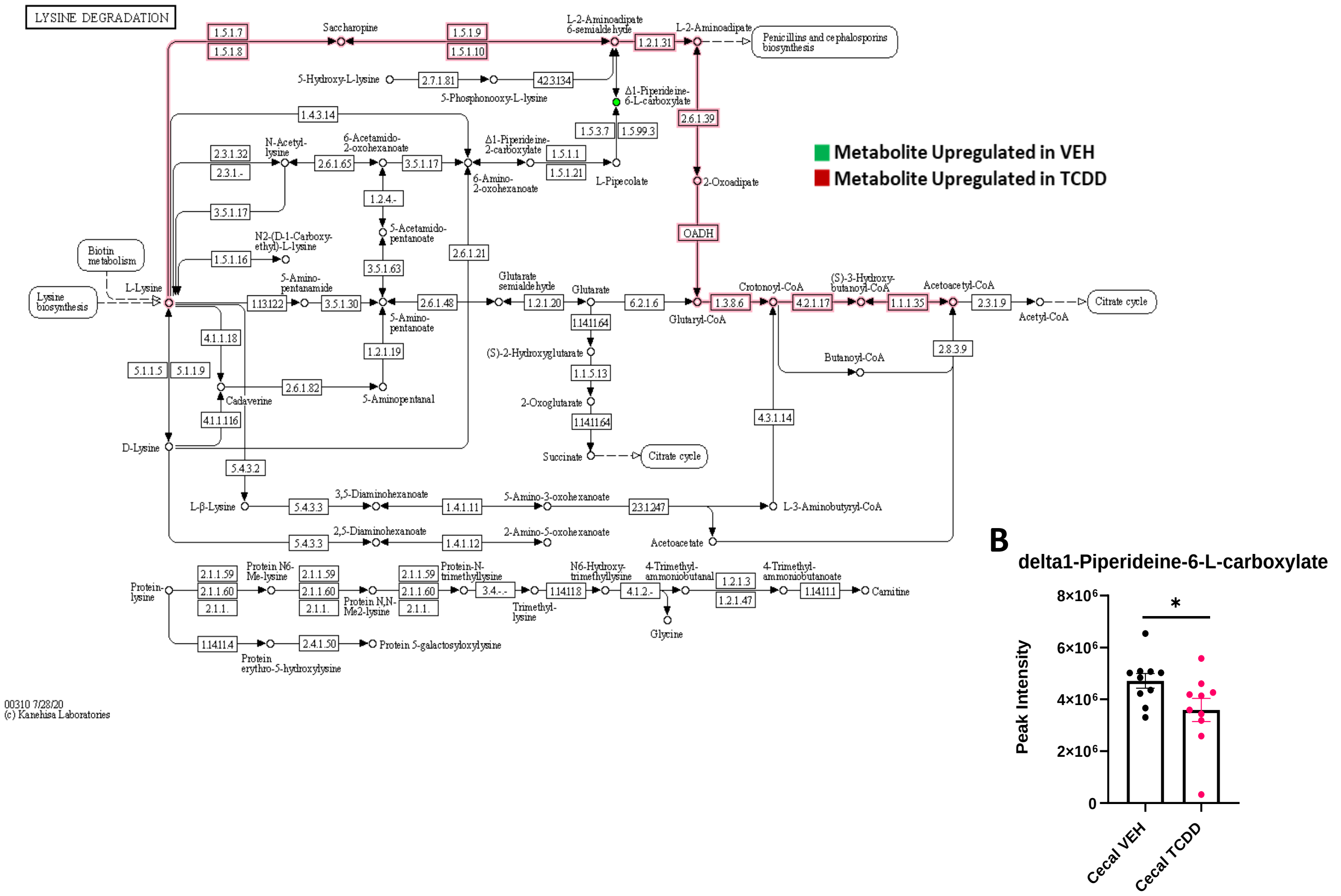

Figure S9. Lysine Degradation in the Cecal Contents. (A) Lysine degradation KEGG pathway illustrating metabolic networks including metabolites denoted by circles and associated enzymes denoted by squares. Significant metabolites are indicated by a solid node that correlates with upregulation in the TCDD (Red) or VEH (Green) exposed mice. Pathway sections and components highlighted by a red outline indicate likely enzymes and additional metabolites that may be involved in the difference of expression. (B) Bar chart demonstrating abundance of delta1-Piperideine-6-L-carboxylate.

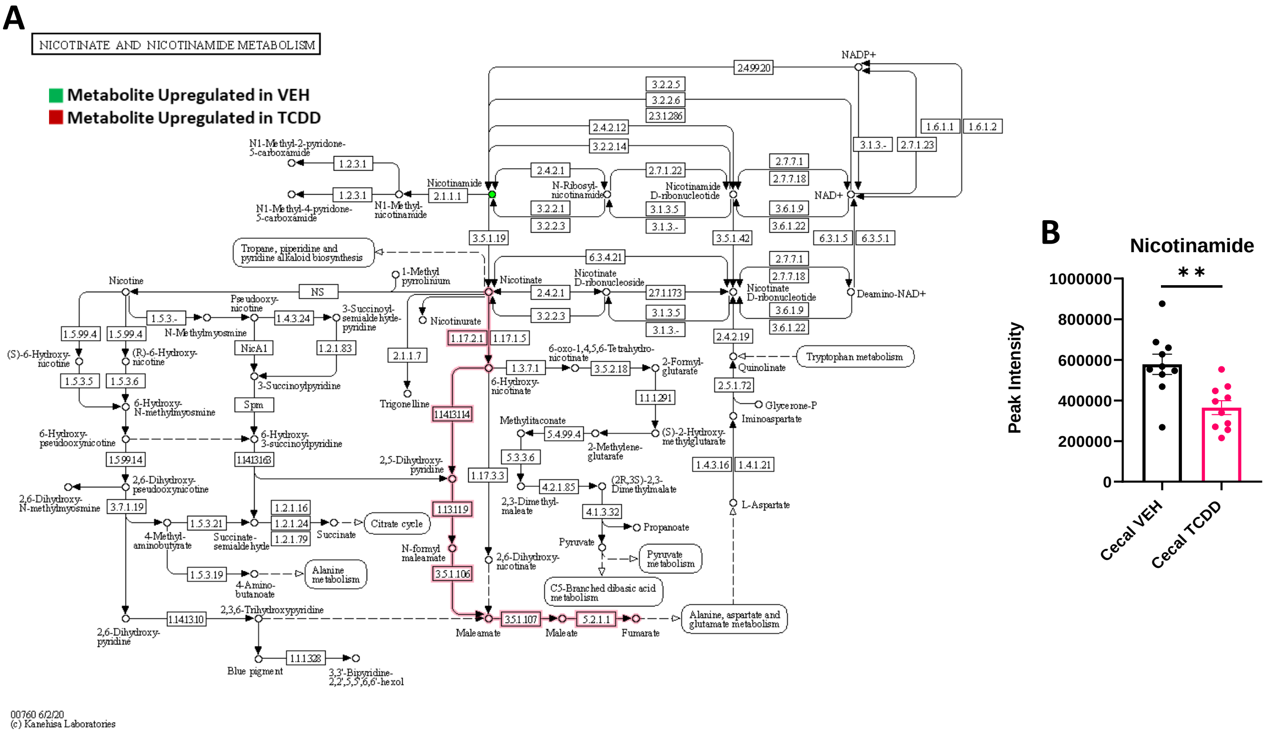

Supplemental Figure S10. Nicotinate and Nicotinamide Metabolism in the Cecal Contents. (A) Nicotinate and nicotinamide metabolism KEGG pathway illustrating metabolic networks including metabolites denoted by circles and associated enzymes denoted by squares. Significant metabolites are indicated by a solid node that correlates with upregulation in the TCDD (Red) or VEH (Green) exposed mice. Pathway sections and components highlighted by a red outline indicate likely enzymes and additional metabolites that may be involved in the difference of expression. (B) Bar chart demonstrating abundance of Nicotinamide.

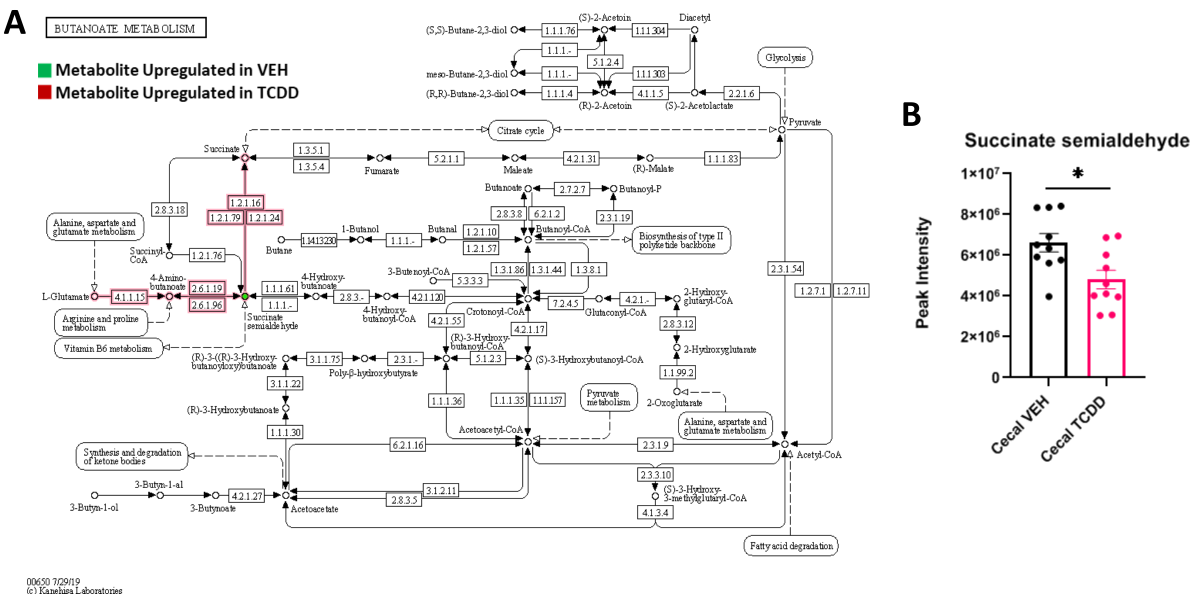

Figure S11. Butanoate Metabolism in the Cecal Contents. (A) Butanoate metabolism KEGG pathway illustrating metabolic networks including metabolites denoted by circles and associated enzymes denoted by squares. Significant metabolites are indicated by a solid node that correlates with upregulation in the TCDD (Red) or VEH (Green) exposed mice. Pathway sections and components highlighted by a red outline indicate likely enzymes and additional metabolites that may be involved in the difference of expression. (B) Bar chart demonstrating abundance of Succinate Semialdehyde.
